# Two Cases of Q Fever in Pregnancy, including Management of the Newborn, Australia

**DOI:** 10.3201/eid3201.251048

**Published:** 2026-01

**Authors:** Robyn Silcock, Robert Horvath, Su May Chew, Clare Nourse

**Affiliations:** Pathology Queensland, Herston, Queensland, Australia (R. Silcock, R. Horvath); Queensland Children’s Hospital, Brisbane, Queensland, Australia (R. Silcock, C. Nourse); University of Queensland, Brisbane (R. Silcock, C. Nourse); Q Fever Interest Group, Brisbane (R. Silcock, R. Horvath, S.M. Chew, C. Nourse); Prince Charles Hospital, Brisbane (R. Horvath); Toowoomba Hospital, Toowoomba, Queensland, Australia (S.M. Chew)

**Keywords:** *Coxiella burnetii*, bacteria, zoonoses, Q fever, pregnancy, breastfeeding, newborn management, Australia

## Abstract

Optimal management of the birthing parent with Q fever in pregnancy and of the infant has not been established. *Coxiella burnetii* expresses a tropism for the placenta; resulting infection can potentially lead to spontaneous abortion and fetal demise. Although evidence around preventing transmission and infection in the peripartum and postpartum period is lacking, reports of healthy babies born to mothers with acute or chronic Q fever in pregnancy are increasing. Historically, many clinicians have recommended against breastfeeding in this setting because of a theoretical risk for bacterial transmission through breastmilk. We discuss 2 women in Australia who had Q fever in pregnancy, focusing on the peripartum period and infant management. Breastfeeding was encouraged in both cases. Both infants were born healthy and at term and have demonstrated no serologic or clinical evidence of Q fever infection in the first year of life.

Optimal management of the birthing parent with Q fever in pregnancy and of the newborn infant has not been established. *Coxiella burnetii*, the causative bacteria for Q fever, has a tropism for the placenta in humans and other mammals (*1*); infection can result in spontaneous abortion and fetal demise. However, several studies have reported healthy, unaffected infants born to mothers with a diagnosis of acute or chronic Q fever in pregnancy (*2*–*7*). Published evidence is lacking to guide best practices for managing such infants. Evidence is currently best gleaned from the occasional published case report of Q fever in pregnancy, despite most having no, or minimal, discussion of infant management. Many experts recommend against breastfeeding (*8*,*9*), although the evidence base for that recommendation remains theoretical (*1*). 

In this article, we discuss 2 women in Australia in whom Q fever was diagnosed in pregnancy, with particular focus on the peripartum period and management of the infant. Breastfeeding was encouraged for both newborn infants. Written and verbal parental consent for the publication of this case report was obtained. This study was approved by the Children’s Health Queensland Hospital and Health Service Human Research Ethics Committee.

## Case 1

A 28-year-old primiparous, previously healthy woman was admitted to hospital with fevers and headaches at 10 weeks’ gestation. She was prescribed 14 days of empiric doxycycline (safe for use during the first trimester); however, diagnosis of Q fever was not made until seroconversion was noted at 24 weeks’ gestation. Retrospective testing of a blood sample from 10 weeks’ gestation detected *C. burnetii* DNA through PCR; results of serologic testing were initially nonreactive (Table 1).

**Table 1:** Serologic and PCR monitoring for case 1 during pregnancy, at delivery, and during the first 12 months postpartum in study of Q fever in pregnancy, including management of the newborn, Australia*

Time	Maternal phase 2 IgM	Maternal phase 2 IgG (IFA)	Maternal phase 1 IgG (IFA)	Infant phase 2 IgM (EIA)	Infant phase 2 IgG (IFA)	Infant phase 1 IgG (IFA)	Infant blood PCR	Maternal blood PCR	Breastmilk PCR
10 weeks’ gestation	Neg	<10	–	–	–	–	–	Detected	–
29 weeks’ gestation	320	5,120	2,560	–	–	–	–	ND	–
At delivery	160	2,560	1,280	Neg	NA	NA	ND	Detected	ND
6 weeks postpartum	320	10,240	10,240	Neg	>1,280	640	ND	ND	ND
4 months postpartum	80	10,240	10,240	Neg	>1,280	320	ND	ND	ND
7 months postpartum	80	20,480	5,120	Neg	160	40	ND	ND	ND
12 months postpartum	80	20,480	5,120	Neg	80	<10	ND	ND	ND

*EIA, enzyme immunoassay; IFA, immunofluorescence assay; NA, not available (not tested); ND, not detected; neg, negative

The woman lived in an urban suburb of Brisbane, Queensland, Australia, with her sister, brother-in-law, 3 nephews, a dog, and a pet lizard. She had not traveled overseas nor had any contact with cattle or sheep farms. A few months before conception, she had commenced work in a pet food cannery. Although Q fever vaccination had been recommended given occupational risk (livestock exposure through pet food), she had not yet been immunized.

Upon retrospective diagnosis of Q fever, trimethoprim/sulfamethoxazole (cotrimoxazole) was prescribed for the period of 25–32 weeks’ gestation, according to local and international recommendations (*10*,*11*). Her routine fetal ultrasound scan at 20 weeks’ gestation found no morphologic abnormalities. A third trimester scan reported mild femur length shortening but was otherwise unremarkable. Third trimester blood tests confirmed the mother’s seroconversion to Q fever (Table 1).

The woman gave birth to a healthy baby boy at 39 weeks, 6 days of gestation by spontaneous vaginal delivery. Birth weight was 2,560 g (3rd percentile). The infant required a 24-hour admission to the special care nursery for hypoglycemia. Of note, at the time of delivery, results of PCR testing of the mother’s blood for *C. burnetii* were positive, and serologic titers in the postpartum period increased.

Placental histopathology revealed acute chorionitis and chorionic vasculitis, noting some unusual features and multinucleate giant cells in the inflammatory infiltrate and focal but prominent necrosis of subchorionic inflammatory infiltrate. Focal changes of chronic villitis were also seen. There was no necrotizing villitis nor placental abscess formation, which differed from previous cases of Q fever placentitis seen in Queensland (L. Taege, pers. comm., pathology report, 2023 Jun 30). *C. burnetii* was detected by PCR from the placenta but was not detected from either cord blood or breastmilk.

Breastfeeding was encouraged in the setting of regular clinical review and serologic and PCR testing of mother, infant, and breastmilk (Table 1). The infant was exclusively breastfed until 6 months of age and continued breastfeeding into the second year of life after introduction of solids. The baby has remained well, with growth parameters tracking on the 80th percentile, and has shown no clinical signs of Q fever infection. Although the infant showed high IgG titers at 6 weeks postpartum, which might represent infection, the titers waned over time, more in keeping with transplacental transfer of maternal IgG. Furthermore, at birth, phase 2 results of IgM serologic testing were nonreactive and remained nonreactive when tested on 4 occasions in the first 12 months of life.

The mother’s echocardiogram 3 months postpartum was normal. Because results of Q fever serologic testing remained strongly positive with a chronic infection profile (Table 1), treatment with doxycycline was recommended. The patient, however, elected not to begin therapy postpartum. Further imaging evaluations, such as fludeoxyglucose-18 positron emission tomography or computer tomography, were not conducted. The mother was advised to contact her infectious diseases physicians before future pregnancies because of the risk for repeat placental infections.

## Case 2

A 30-year-old woman, gravida 6, para 4, tested positive for Q fever on serologic tests and PCR of blood taken at 8 weeks’ gestation, discovered as part of a public health investigation. Two months earlier, she had aided in the delivery of a calf, where known Q fever exposure had occurred. The woman lived on a dairy farm with her children and partner. Pathologic testing at that time revealed reactive phase 2 and phase 1 IgG and IgM (Table 2). Retrospective review of available serologic tests found similar results from 4 months earlier (Table 2). Of note, Q fever DNA was detected in both blood samples by PCR, although a dedicated blood tube was not obtained in either case. Q fever serology from 10 years earlier was negative. It was thus deemed that the patient, at the time of diagnosis, had chronic/persistent focalized Q fever infection in pregnancy, although the exact timing of infection was unclear. The patient was started on doxycycline during the first trimester until 13 weeks, then took cotrimoxazole with folic acid supplementation from 14 to 34 weeks’ gestation. Serologic tests for Q fever remained positive throughout her pregnancy, but PCR of blood did not detect Q fever on 3 other occasions.

**Table 2:** Serologic and PCR monitoring for case 2 during pregnancy, at delivery, and during the first 9 months postpartum in study of Q fever in pregnancy, including management of the newborn, Australia*

Time	Maternal phase 2 IgM	Maternal phase 2 IgG (IFA)	Maternal phase 1 IgG (IFA)	Infant phase 2 IgM (EIA)	Infant phase 2 IgG	Infant phase 1 IgG	Infant blood PCR	Maternal blood PCR	Breastmilk PCR
3 months preconception	320	320	40	–	–	–	–	Detected	–
8 weeks’ gestation	80	160	160	–	–	–	–	Detected	–
14 weeks’ gestation	160	320	640	–	–	–	–	ND	–
20 weeks’ gestation	160	>1,280	>1,280	–	–	–	–	ND	–
32 weeks’ gestation	80	320	640	–	–	–	–	ND	–
At delivery	80	80	320	Neg	320 (IFA)	80 (IFA)	ND	ND	ND
6 weeks postpartum	40	80	160	NA	NA	NA	NA	ND	ND
4 months postpartum	80	80	80	Neg	EIA neg	EIA neg	ND	ND	ND
6 months postpartum	80	80	160	Neg	EIA neg	EIA neg	ND	ND	ND
9 months postpartum	80	80	320	NA	NA	NA	NA	NA	NA

*EIA, enzyme immunoassay; IFA, immunofluorescence assay; NA, not available (not tested); ND, not detected; neg, negative; –, not applicable.

The pregnancy was otherwise uncomplicated, and no morphologic abnormalities were identified by ultrasound. A healthy baby boy was born by induced vaginal delivery at 37 weeks’ gestation with a birth weight of 2,510 g (2nd percentile). Q fever was not detected from the placenta or breastmilk by PCR. Placental histology showed no signs of acute or chronic infection. Breastfeeding was encouraged.

In the 9 months postpartum that were documented, the mother’s phase IgG has remained elevated, 1:320 at most recent testing (Table 2). The baby has remained well; growth parameters have tracked on the 10th to 25th percentile, and no serologic evidence of Q fever acquisition has been noted (IgM nonreactive, IgG nonreactive by 6 months of age [Table 2]). *C. burnetii* PCR of breastmilk was negative on 4 occasions from birth until 6 months postpartum. The mother chose to cease breastfeeding at 8 months postpartum.

## Literature Review

*C. burnetii* is found in all areas of the world except New Zealand and Antarctica (*12*). At least half of Q fever infections are asymptomatic (*13*). Rarely, a persistent focalized infection (also known as chronic Q fever) develops (*12*,*13*). Persistent or chronic infection is more common during pregnancy because of the bacteria’s ongoing replication within the placenta (*14*). Population-based seroprevalence studies have shown mixed results when evaluating the effects of Q fever infection on obstetric outcome (*15*).

The management of Q fever perinatally is beset by lack of evidence and difference of opinion. No consensus guidelines exist for the management of Q fever in pregnant women or newborns, although some national guidelines comment specifically on the treatment of Q fever in pregnancy (*10*,*14*). Several case series and case reports of Q fever in pregnancy have been published. The largest case series of 53 patients from France over a 15-year period found obstetric complications in 37 (69.8%) women, most commonly intrauterine growth retardation, intrauterine fetal death, and premature delivery (*16*). Another large series from France involving 29 women reported obstetric complications in 66% of women (*9*). Both series reported improved obstetric outcomes in those women prescribed antimicrobial therapy (cotrimoxazole or roxithromycin) for at least 5 to 10 weeks before delivery. However, a marked selection bias is present in those studies because Q fever is often only diagnosed at the time of fetal demise or other obstetric complication, especially in persons who did not receive antimicrobial therapy (*9*,*16*).

Case series from the Netherlands and Germany during Q fever outbreaks present a more optimistic picture. Munster et al. (*17*) compared 183 seropositive women to 1,046 seronegative women during a large outbreak in the Netherlands and found no difference in preterm delivery, birth weight, size for gestational age, or perinatal mortality between the groups. A smaller series from Germany involved 11 women during 2 distinct outbreaks. Infected women were offered a variety of treatment (cotrimoxazole, macrolide, sulfadiazine, and pyrimethamine or a combination thereof), and 2 women received no treatment (*2*). Of those 11 pregnancies, 1 maternal death occurred (reported as unrelated), 1 child was born with syndactyly (whose mother had received clarithromycin), and 1 late preterm delivery at 35 weeks occurred. The other 8 infants were born at term and healthy (*2*).

Antimicrobial treatment in women with Q fever infection in pregnancy is complicated by concerns about potential maternal and neonatal toxicity. For both reported women, treatment was initiated upon diagnosis. Because of concern about congenital anomalies and miscarriage, trimethoprim/sulfamethoxazole was avoided in the first trimester, and in both cases doxycycline was prescribed. Doxycycline is considered safe in pregnancy until 18 weeks’ gestation. Beyond that time, doxycycline is not recommended because of concerns around bone growth inhibition and discoloration of deciduous teeth of the newborn, complications that were observed with the use of earlier tetracyclines. However, despite concerns, evidence is lacking that doxycycline causes tooth discoloration or bone growth inhibition, and doxycycline is increasingly used in children (*18*,*19*). With regard to cotrimoxazole in pregnancy, concerns persist that it could be linked to hemolytic anemia and jaundice in the newborn after maternal treatment close to delivery, but evidence does not support that link (*20*). Although current US Centers for Disease Control and Prevention guidelines recommend that physicians consider ceasing cotrimoxazole at 32 weeks’ gestation, many physicians, including ourselves, recommend continuing to 36 weeks (*15*) or until delivery (*14*). Of note, the cessation of cotrimoxazole at 32 weeks in case 1 might have contributed to blood PCR positivity at the time of birth and subsequent higher maternal serologic titers. Although doxycycline has been shown to have superior anti-*Coxiella* activity than cotrimoxazole (*21*), until further safety data are available on doxycycline’s use during the second and third trimesters, we recommend doxycycline until week 16 followed by cotrimoxazole until delivery.

Several other case reports have been published after Q fever infection in pregnancy (Table 3). In the absence of universal or prospective Q fever testing, published case series and case reports might have a selection bias, because relatively asymptomatic Q fever infection in an uncomplicated pregnancy is unlikely to be assessed for Q fever infection.

Acknowledging the limitations of seroprevalence studies and the selection bias of case reports and series, we have not found any evidence of congenital Q fever syndrome. Although fetal demise can occur and there have been 2 documented cases of Q fever detection in aborted fetuses confirming placental transmission (*32*,*33*), babies born alive after Q fever infection in pregnancy show no signs or sequelae of in utero infection.

Similarly, no definitive case of neonatal or infant Q fever has ever been reported. Four cases of infant Q fever have been described internationally; however, all of those reports have a likely alternative diagnosis or represent transplacental transfer of antibodies (*34*–*37*). Of note, no infant cases of Q fever were reported in the large, well-documented outbreaks in the Netherlands (*38*,*39*).

Despite the lack of reports of confirmed Q fever in neonates or infants, concerns around Q fever transmission through breastmilk are often cited (*6*,*8*). *C. burnetii* DNA has been detected by PCR in dairy milk, although transmission through ingestion of unpasteurized dairy products has not been conclusively demonstrated (*12*,*40*). *C. burnetii* has also been identified in human milk. A 1981 paper from India reported 5 of 97 samples of human milk tested were found to be positive for *C. burnetii* or its antibody (*41*). A further study from India in 1986 reported 22 of 153 human milk samples demonstrated *C. burnetii* antibodies; 4 of the positive samples then demonstrated the presence of the bacteria by specific seroconversion in guinea pigs (*42*). On the basis of those reports, in 1990, Langley concluded, “*C. burnetii* can be excreted in human milk” (*1*). This review went on to describe 1 previously published case of a 9-month-old baby in Scotland who died of sudden infant death syndrome in 1983. In the postmortem examination, high Q fever antibodies were identified, and acquisition of Q fever through breastmilk was speculated but not confirmed (*36*). The theoretical risk for breastmilk transmission of Q fever has subsequently been used to recommend that women who have had Q fever during pregnancy do not breastfeed (*8*).

Infant feeding practices are often not discussed in case reports of perinatal Q fever. Among the numerous published case series and studies of Q fever in pregnancy (Table 3), only 5 commented on infant feeding practices (*4*,*7*,*23*,*27*,*43*); breastfeeding was permitted in just 1 recent case in Australia (*7*). The authors report the baby was healthy and asymptomatic at the time of writing (4 months of age) (*7*). A case series from Limoges, France, references “women who breastfed against recommendations” (*9*), and a series from Germany alludes to cessation of breastfeeding upon detection of C. *burnetii* by PCR in breastmilk (*2*). Neither report specifically commented on follow-up of the breastfed infant(s) (*2*,*9*).

The benefits of breastfeeding to both mother and baby have become more evident in recent decades (*44*–*46*). Breastfeeding in mothers living with other infectious diseases, such as HIV, is becoming more widely accepted and supported in many countries (*47*). Given the paucity of evidence to recommend against breastfeeding in Q fever infection, in both of the cases reported in this study, breastfeeding was encouraged in accordance with the mothers’ preferences. No transmission of Q fever occurred; both infants have tested negative by blood PCR on multiple occasions and have shown no signs of seroconversion. Results of PCR testing of breastmilk for *C. burnetii* were negative on every occasion (Table 1, Table 2).

Infection control precautions at the time of delivery to prevent potential mother-to-child transmission or nosocomial transmission to staff and other patients are key (*32*,*48*). *C. burnetii* is aerosolized, and the placenta carries high bacterial loads even after antenatal treatment. Airborne precautions in the delivery suite and operating theater are recommended. In both cases described, the use of interventions such as scalp electrodes was permitted as clinically indicated, the mother and infant were not separated, and the infant was washed after delivery and then managed with standard precautions. *C. burnetii* is highly resistant to inactivation by standard disinfectants. The US Centers for Disease Control and Prevention recommends cleaning with Micro-Chem Plus (a dual-quaternary ammonium/detergent compound) (National Chemical Laboratories, https://www.nclonline.com), a 1:100 dilution of household bleach, or 1% Virkon S treatment (*14*).

After the birth of an infant to a parent with Q fever in pregnancy, we recommend histologic examination and PCR testing of the placenta; clinical, serologic, and PCR monitoring of mother and infant for a period of 6 months to 1 year; and, if feasible, serial PCR testing of breastmilk. Our second case also demonstrates the potential for Q fever to develop chronicity or recrudescence after preconception acute infection, likely reflecting *Coxiella burnetii*’s tropism for uterine tissue. This phenomenon has been previously described in 1 woman during the outbreak in the Netherlands (Table 3) (*22*). As such, close observation for Q fever in pregnancy should also be recommended for any cases of acute Q fever in the period 3–6 months before conception. Reactivation of Q fever in subsequent pregnancies has also been described (*33*).

In conclusion, we report 2 women with Q fever in pregnancy, 1 infected in the first trimester and 1 likely infected 3–6 months before conception with persistent focal disease evident through pregnancy. Both women received antimicrobial therapy (doxycycline and cotrimoxazole) until 32–34 weeks’ gestation. In both cases, healthy, albeit small, infants were born at term with no evidence of long-term sequelae or in utero infection. Both infants were breastfed for >6 months without transmission of Q fever. Although transmission precautions at the time of delivery remain key, our experience, as well as review of the literature, provides reassurance for women who wish to breastfeed after Q fever infection during pregnancy. Given the rarity and paucity of strong scientific evidence, we would advocate that all pregnant persons with Q fever infection be referred to an expert group.

## Figures and Tables

**Table:** ​**Table 3.** Published cases of Q fever infection in pregnancy reviewed for study of Q fever in pregnancy, including management of the newborn, Australia*

**Mother’s age, country (reference)**	**Gestation**	**Treatment**	**Outcome**
42 y, the Netherlands (*22*)	Shortly before conception; steep increase in IgG phase I and IgG phase II and PCR serum positive at 25 weeks’	Cotrimoxazole (allergy so changed to erythromycin)	Induction of labor at 38 weeks; healthy baby, birth weight 3,850 g; amniotic fluid and placenta PCR positive; newborn blood PCR negative
29 y, Slovenia (*3*)	Seroconversion found at 9 weeks; febrile illness 10 days before conception	Azithromycin for 6 d at 9 weeks’ gestation	Spontaneous vaginal delivery at term; birth weight 3,500 g; amniotic fluid and placenta PCR negative
39 y, Australia (*4*)	Fever at 7 weeks; seroconversion at 9 weeks	Cotrimoxazole from 9 to 36 weeks’ gestation	Spontaneous vaginal delivery at term; birth weight 3,600 g; placenta, blood, breastmilk PCR negative
27 y, Germany (*23*)	Acute Q fever at 7 weeks; retrospective diagnosis at 19 weeks	Erythromycin from 25 to 26 weeks’ gestation; rifampin + clarithromycin from 26 weeks through delivery	Delivery at 30 weeks; birth weight 3,900 g
28 y, Spain (*5*)	14 weeks	No treatment	Delivery at 36 weeks; healthy baby, birth weight 2,125 g
26 y, United Kingdom (*24*)	14 weeks	No treatment	Intrauterine fetal demise at 25 weeks; *C. burnetii* detected on placental stains
28 y, Israel (*25*)	22 weeks’ gestation; fever since 16 weeks; acute Q fever on serology	Erythromycin and rifampin from 22 to 30 weeks’ gestation	Premature labor at 30 weeks; birth weight 1,300 g; baby treated for 14 d with rifampin + erythromycin; complete recovery
29 y, Israel (*26*)	21 weeks’ gestation; fevers since 17 weeks; chronic Q fever on serology	Erythromycin at 21 weeks’ gestation, then tetracycline from 22 weeks gestation until induction at 28 weeks	Induced at 28 weeks; birth weight 1,000 g; placenta necrotic; *C. burnetii* isolated; baby not infected; yellow teeth
34 y, Spain (*27*)	21 weeks’ gestation; febrile; acute Q fever on serology	Cotrimoxazole from 21 weeks’ gestation until term	Delivery at 40 weeks; healthy baby, birth weight 2,930 g; formula fed
18 y, Spain (*28*)	Fevers at 19 weeks’ gestation; seropositive 1 month later	Clarithromycin commenced at 20 weeks’ gestation; duration not specified	Delivery at 40 weeks; healthy baby
34 y, Israel (*29*)	Pyrexia of unknown origin at 24 weeks’ gestation; 26 weeks abruption	No treatment	Viable baby delivered; birth weight 967 g; PCR Q fever positive
27 y, Israel (*30*)	26 weeks’ gestation; 3-week history of fevers	Doxycycline commenced at 26 weeks’ gestation, continued until IUFD at 27 weeks	Intrauterine fetal demise at 27 weeks
29 y, Australia (*7*)	29 weeks’ gestation	Cotrimoxazole from 29 to 30 weeks’ gestation; clarithromycin from 31 weeks until term (rash with cotrimoxazole)	Medical induction at 39 weeks; healthy baby; amniotic fluid, fetal blood, and placenta PCR negative
26 y, United Kingdom (*31*)	28 weeks’ gestation (acute Q fever seroconversion between 16 and 29 weeks)	Ciprofloxacin from 29 weeks’ gestation until induction at 32 weeks	Induced at 32 weeks; healthy baby
34 y, Israel (*29*)	Fevers at 29 weeks’ gestation	No treatment	Placental abruption; delivered at 31 weeks; healthy baby; birth weight 1,514 g; placenta PCR positive
22 y, Australia (*6*)	Fevers at 28 weeks’ gestation; acute Q fever on serology	Cotrimoxazole from 29 weeks’ gestation until term	Spontaneous vaginal delivery at 40 weeks; *C. burnetti* detected on PCR of placenta, not detected in breastmilk; patient well

*Cotrimoxazole, trimethoprim/sulfamethoxazole.
